# Functional analysis of type II chalcone isomerase (*CHI*) genes in regulating soybean (*Glycine max L*.) nodule formation

**DOI:** 10.1080/21645698.2025.2486280

**Published:** 2025-03-31

**Authors:** Xinyue Wang, Jingwen Li, Yuxue Zhou, Jinhao Zhang, Le Wang, Yajing Liu, Xuguang Yang, Hongshuang Han, Qingyu Wang, Ying Wang

**Affiliations:** College of Plant Science, Jilin University, Changchun, China

**Keywords:** Soybean, root nodulation, chalcone isomerase, isoflavones, biological nitrogen fixation, leguminous plants

## Abstract

Biological nitrogen fixation (BNF) is the most cost-effective and environmentally benign method for nitrogen fertilization. Isoflavones are important signaling factors for BNF in leguminous plants. Whether chalcone isomerase (*CHI*), the key enzyme gene in the flavonoid synthesis pathway, contributes to soybean (*Glycine max*) nodulation has not yet been fully clarified. In the present study, we identified the functions of three types of *GmCHI* for BNF using a hairy root system. The results showed that *GmCHI1A* and *GmCHI1B1* positively increased nodulation while *GmCHI1B2* did not, with the *GmCHI1A* gene having a greater effect than *GmCHI1B1*. Meanwhile, the daidzein and genistein contents were significantly increased in composite plants overexpressing *GmCHI1A* and reduced in composite plants, thus interfering with *GmCHI1A*. However, overexpression of *GmCHI1B1* significantly increased the content of glycitein but not daidzein, genistein content implied that homologous genes exhibit functional differentiation. These results provide a reference for subsequent studies on improving nitrogen fixation in soybeans and providing functional genes for the improvement of new varieties.

## 1. Introduction

Nitrogen is a critical element for all organic life forms, and symbiotic nitrogen fixation contributes approximately 20 million tons annually.^1^ Most plants need to take up nitrogen from the soil, although more than 90% of the nitrogen in the soil is organic and cannot be directly absorbed and used by plants.^2^ Biological nitrogen fixation (BNF) is the most cost-effective and environmentally benign method of nitrogen fertilization, and high levels of nitrogen fixation increase legume productivity and contribute to sustainable agriculture.^3^ Among the various nitrogen fixation processes, symbiosis between rhizobia and legumes is considered the most effective BNF system, accounting for more than half of the global nitrogen fixation.^4^ The development of nodules in legumes is a highly intricate process that is synergistically regulated at both the physiological and molecular levels by plants and rhizobia. Studies have shown that BNF begins with flavonoid compounds secreted by legume roots, after which rhizobia are attracted to and stimulated to produce nodulation factors.^5^ These nodulation factors are sensed by receptor protein kinases (LysM RLK and LRR RLK) in the roots,^6^ initiating a symbiotic signaling cascade that leads to rhizobial infection and cortical cell division. Eventually, cortical cells divide to form rhizobial progenitors, which develop into mature rhizomes, which execute efficient nitrogen fixation^7^

Flavonoids are important signaling molecules in the symbiosis between rhizobia and legumes,^8^ and they attract rhizobacteria, synthesize nodulation factors, stimulate nodulation symbiosis signal transduction, participate in rhizobial organogenesis, and regulate rhizome formation.^9^ Flavonoids secreted by the root system of legumes are primarily found in soybeans (*Glycine max*), alfalfa (*Medicago sativa*), peas (*Pisum sativum*), and wood beans (*Cajanus cajan*).^10^ As common flavonoids secreted by the root systems of legumes, soy isoflavones have been demonstrated to possess physiological activities, including the induction of root nodule formation.^11^

Isoflavone biosynthesis begins with the deamination of phenylalanine, which is catalyzed by phenylalanine ammonia-lyase (PAL) to form cinnamic acid. Cinnamic acid is then hydroxylated by cinnamate-4-hydroxylase (C4H) to form p-coumaric acid, which is subsequently activated by 4-coumarate-CoA ligase (4CL) to produce p-coumaroyl-CoA, a common precursor in flavonoid biosynthesis.^12^ In the later stages of this pathway, p-coumaroyl-CoA is converted into isoliquiritigenin or naringenin chalcone by chalcone reductase (CHR) and chalcone synthase (CHS). These chalcones are then transformed by chalcone isomerase (CHI) into liquiritigenin or naringenin. Finally, isoflavone synthase (IFS) and 2-hydroxyisoflavone dehydratase (HID) catalyze the formation of isoflavonoid aglycones, such as daidzin, genistein, and glycitein.^13,14^ This biosynthetic pathway is predominantly found in legumes.^15^ The two principal isoflavonoids in soybeans are daidzein and genistein, which play pivotal roles in the formation of nodules and regulation of nodule ratios.^16^ Isoflavones are key molecules in symbiotic signaling between legumes and rhizobia that induce the production of nodulation factors (Nod factors) by rhizobia, which in turn initiates nodule formation.

CHI is the second rate-limiting and first reported enzyme in flavonoid biosynthesis. It catalyzes intramolecular cyclization reactions converting bicyclic chalcones into tricyclic (2S)-flavanones.^17^ Expression levels of *CHI* in plants have a crucial positive effect on flavonoid metabolism.^18^ Previous studies have divided the *CHI* family genes into four different types (types I-IV). Both type I and type II proteins are CHIs that present enzymatic activity to catalyze the stereospecific isomerization of chalcones to yield their corresponding flavanones.^19^ Notably, this cyclization reaction can also proceed spontaneously, although greater catalytic efficiency (107-fold) occurs with the aid of these CHIs. Type I *CHIs* are widely present in vascular plants and involved in the production of (2S)-naringenin (5,7,40 -trihydroxyflavanone) by exclusively isomerizing naringenin chalcone (4,20,40,60 -tetrahydroxychalcone). Type II *CHIs* appear to be specific to legumes and are primarily responsible for isoflavonoid production.^20^ Compared with these type I and II *CHIs*, type III and type IV *CHIs* are also found in plants but do not exhibit CHI activity due to the substitution of several catalytic core residues.^21^

The soybean CHI family also contains four subfamilies: Type I: *GmCHI2*; Type II: *GmCHI1A, GmCHI1B1, GmCHI1B2;* Type III: *GmCHI3A1, GmCHI3A2, GmCHI3B1, GmCHI3B2, GmCHI3C1, GmCHI3C2*; and Type IV: *GmCHI4A, GmCHI4B*.^22,23^ Type I *CHIs* is expressed predominantly in floral tissues, whereas Type II *CHIs* is root-specific and regulates enzymatic activity in isoflavonoid synthesis.^24^

Therefore, in this study, we investigated the effects of type II *CHIs* on soy isoflavone content and nodulation. Composite plants transformed with *GmCHI1s* using a soybean hair-rooting technology system were obtained, the main functional genes were screened, and the role of *GmCHI1s* in the symbiotic nitrogen fixation process of soybean rhizobia was elucidated.

## 2. Materials and Methods

### 2.1. Plant Materials and Plant Growth Conditions

Soybean cultivar Williams 82 was grown in an artificial climate chamber at Jilin University, Changchun, China, under a 16 h light (25°C)/8 h (22°C) diurnal cycle with 75% relative humidity. The roots, stems, leaves, flowers, and mature seeds were collected for cloning and expression analysis of *GmCHI1s* (*GmCHI1A*, *GmCHI1B1* and *GmCHI1B2*). All collected samples were immediately plunged in liquid N_2_ and then were stored at − 80°C prior to RNA isolation. Three independent biological replicates (at least ten replicates) were used for RNA extraction.

### 2.2. Cloning of GmCHI1s Genes

TRIzol reagent was used to extract root RNA from Williams 82. RNA solution was reverse transcribed into cDNA using a PrimeScript™ II 1st Strand cDNA Synthesis Kit (Takara, Changchun, China)^25^. The open reading frames (ORFs) of *GmCHI1s* were amplified from root cDNA using primers (Table S1). Polymerase chain reaction (PCR) was performed as follows: 94°C for 8 min, followed by 30 cycles of 94°C for 30 s, 50/58°C for 30 s, and 72°C for 1 min followed by a final extension at 72°C for 8 min. The amplified fragments were cloned into the pMD-18T cloning vector (TaKaRa, Changchun, China) and sequenced for confirmation (Kumei Technology Co., Ltd., China).

### 2.3. Bioinformatics Analysis of the GmCHI1s Gene Sequences

Gene sequences of *GmCHI1s* were searched and compared using the NCBI database (https://www.ncbi.nlm.nih.gov/). *GmCHI1s* protein sequences were analyzed using the ProtParam software (http://web.expasy.org/protparam/). The amino acid sequences of *GmCHI1s* genes were compared and analyzed using the DNAMAN software. A phylogenetic tree was constructed using the neighbor-joining method in MEGA6.0 software. Bootstrapping values were presented from 1000 repeated calculations, and bootstrap values of > 70% were recommended.

### 2.4. Construction of Hairy Root Expression Vector

Using the double-enzyme digestion method, the *GmCHI1s* genes were recombined to construct an overexpression vector (pCHF1301) and interference vector (pFGC5941). The overexpression vector pCHF1301 was digested with the restriction endonucleases bamH1 and Pst1, and the interference vector pFGC5941 was digested with the restriction endonucleases Nco1 and Asc1. Specific primers were designed, and the corresponding cleavage junction was inserted at the 3’ end of the primers, and the plasmid pMD18-T-*GmCHI1s* containing the complete coding sequence of the *GmCHI1s* genes was used as a template for PCR amplification. The product was purified, recovered, ligated with the pMD18-T vector, and sequenced for confirmation (Kumei Technology Co. Ltd., China). The correct fragment was ligated into the appropriate vector using T4 ligase. The recombinant plasmid was then transformed into *Escherichia coli* (DH5α) and verified by colony PCR. The correct recombinant plasmids pCHF1301-*GmCHI1s* and pFGC5941-*GmCHI1A* were transformed into the *Agrobacterium rhizogenes* strain K599 using the freeze-thaw method (Weigel & Glazebrook, 2006).^26^

### 2.5. GmCHI1s Transformation into Composite Plants

Williams 82 seeds were grown in pre-sterilized wet vermiculite at a depth of 5 cm for germination, 7 day old soybean seedlings were injected at the cotyledonary node locus with *A. rhizogenes* (K599) medium with a recombinant expression vector,^27^ and a null control was established. Hairy roots appeared after two weeks and were transplanted after subtracting the primary roots. The slow-growing rhizobium *Bradyrhizobium japonicum* USDA110 was also cultured at 28°C in Yeast Mannitol Agar (YMA) medium. Approximately 50 mL of a suspension with an optical density of approximately 0.05 to 0.1 at 600 nm (OD_600_) adjusted to 0.08 to 0.1 was applied to each plant.

### 2.6. Phenotyping of Transgenic Plants

Four weeks after rhizobial application, the phenotypic data of hairy roots, nodules, and leaves were collected. The Soil Plant Analysis Development (SPAD) chlorophyll meter and nitrogen content (in mg/g) of the leaves of the soybean composite plants were determined, and the number of hairy roots, length of roots, weight of roots, number of rhizomes, weight of rhizomes, and volume of rhizomes of the composite plant roots were measured. Eight seedlings were analyzed for each transformation vector and control.

### 2.7. Isoflavones Extraction and High Performance Liquid Chromatography (HPLC) Analysis

The fresh weight of soybean composited plant transgenic root samples to approximately 0.5 g was weighed, 80% methanol (analytical purity) extract was added to a mortar and the sample was grinded to a homogenate. Then, the sample was transferred to a 10 mL volumetric flask to set the volume. The sample was ultrasonicated at 50°C (frequency 40 kHz, power 300 W) to assist in extraction for 40 min. Every 10 min, the sample will be taken out of the sample up and down the upside down mixing. Ultrasonic centrifugation was then performed at 12,000 rpm and room temperature for 10 min. After sonication, the supernatant was removed, passed through a 0.45-μm filter membrane to obtain the solution to be tested, and stored at 4°C for testing.

The solvents and distilled water were of HPLC grade and degassed before use. A Phenomenex C18 column (Shimadzu LC-20A, Japan, 150 mm × 4.6 mm, 5.0 μm) was utilized for HPLC. The sample solution (40 µL) was aspirated, and the isoflavone content of each component was determined using liquid chromatography, with three replicates performed for each sample.

### 2.8. Quantitative RT-PCR (qRT-PCR) Analysis of Gene Expression

The *GmCHI1s* gene expression patterns in various tissues were examined using qRT-PCR. The primers used for qRT-PCR are listed in Table S1. For analysis of tissue-specific expression, total RNA was isolated from different organs (seeds, roots, stems, leaves, and flowers) and transgenic hairy roots using RNA plant Plus Reagent (Tiangen, Changchun, China) according to the manufacturer’s protocol. cDNA was reverse transcribed using M-MLV reverse transcriptase (TaKaRa, Changchun, China). The qRT-PCR was performed using SYBR Green I dye (TaKaRa, Changchun, China) on a real-time PCR machine (Applied Biosystems 7500, Foster City, USA). Soybean *GmActin* was used as an internal control.

### 2.9. Statistical Analysis

Samples were subjected to statistical analysis using IBM SPSS19.0 and GraphPad Prism 8.0 software. Data were presented as the mean ± SD. Statistical significance among the treatments was evaluated using one-way analysis of variance (ANOVA) and Student’s t-test. Asterisks or different lowercase letters indicate significant differences according to *p* < .05 or *p* < .01.

## 3. Result

### 3.1. Sequence and Expression Analysis of *GmCHI1s* in Soybean

**Figure 1.**
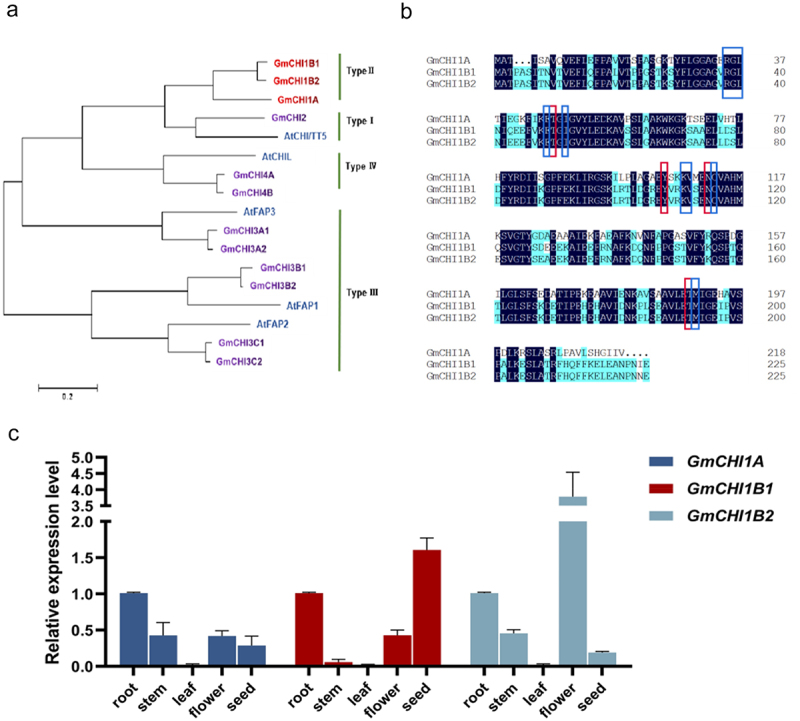
Sequence and expression analysis of *GmCHI1s*. (a) A phylogenetic tree containing *GmChI1s* and 14 other species of CHI proteins was obtained using the neighbor joining method of the MEGA software. (b) Comparison of the protein sequences of *GmChI1s* using DNAMAN software. (c) The expression of *GmChI1s* in roots, stems, leaves, flowers and mature seeds.

*GmCHI1A*, *GmCHI1B1*, and *GmCHI1B2* were cloned by PCR using soybean Williams 82 cDNA as a template. Bioinformatic analysis showed that *GmCHI1A*, *GmCHI1B1*, and *GmCHI1B2* were the most recently identified type II *GmCHIs* (see Figure 1a). *GmCHI1A* contained an entire ORF of 657 bp that encodes 218 amino acids. The ORFs of *GmCHI1B1* and *GmCHI1B2* both contained 681 bp, which encoded 226 amino acids. Conserved structural domains of these three genes showed 87.32% homology. *GmCHI1B1* and *GmCHI1B2* had higher homology in the conserved protein domain, with a value of 94.46%. All three genes retained all residues of the catalytically active sites of the CHI protein, which are related to the catalytic ability of trihydroxychalcone and tetrahydroxychalcone (see Figure 1b).

qRT-PCR was used to detect the expression levels of *GmCHI1s* in the roots, stems, leaves, flowers, and seeds. The results showed that the expression of these three genes was observed in all tested samples. However, their expression patterns differed among organs. The highest level of *GmCHI1A* gene was observed in the roots, which was two times the expression in stems, flowers and seeds, and the lowest expression was observed in the leaves; The highest level of *GmCHI1B1* gene was detected in the seeds, which was 1.5 times the expression in the roots and 3 times of the expression in the flowers, and the lowest expression was observed in the stems and leaves; The highest level of *GmCHI1B2* expression was observed in the flowers, which was 2.5 times higher than that in the roots and 7 times higher than that in the stems. The expression of *GmCHI1A* was higher in the roots than in the other organs, implying that it was a possible that *GmCHI1A* works mainly in roots (see Figure 1c).

### 3.2. Overexpression of *GmCHI1s* in Soybean Resulted in a Differential Increase in Root Nodulation

**Figure 2.**
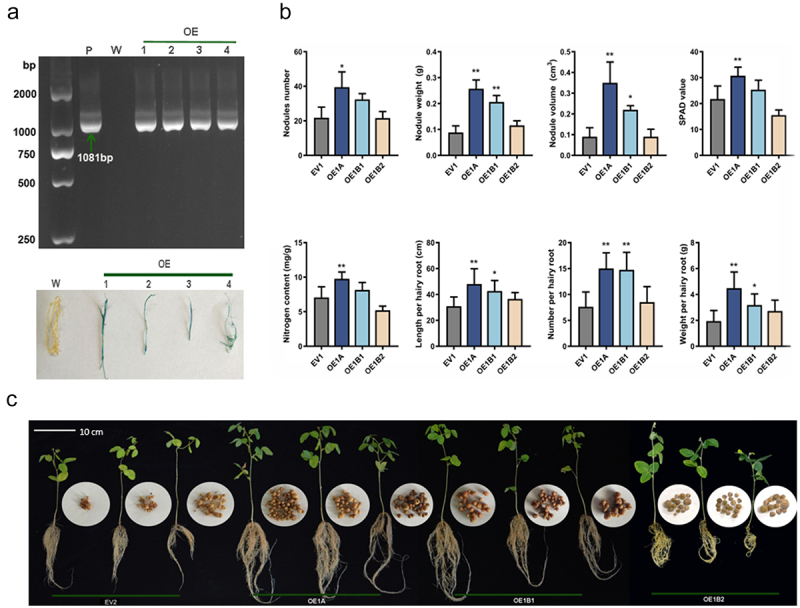
The effect of overexpression of *GmCHI1s* genes on root nodulation formation in soybean. (a) PCR test results of GUS gene and GUS staining results of the overexpressing *GmChI1s* roots (P: positive plasmid; W: negative control; 1: empty pCHF-gus hairy root; 2~4: experimental group pCHF-CHI1s hairy root); (b) phenotypic data statistics of comparing between overexpressing transgenic composite plants and control (** and *indicate significance at *p* ≤ .01 and *p* ≤ .05 respectively). (c) Phenotypic map of soybean overexpressing transgenic composite plants and control.

To determine whether the overexpression of *GmCHI1s* affects nodulation in soybeans, we generated transgenic plants overexpressing *GmCHI1s* using the soybean hairy rooting technique (see Figure 2a). The control consisted of soybeans transformed with an pCHF-1301 empty vector (EV1). The phenotypic characteristics of the composite plants were investigated 30 d after rhizobial infection. The aboveground leaf and belowground root growth statuses of overexpression plants were better than those of the control group. The results showed that overexpressing *GmCHI1s* affected soybean nodulation and growth to varying degrees (see Figure 2c). The nodule number, weight, and volume significantly increased in the roots of the overexpressing *GmCHI1A* (OE1A) plants. Simultaneously, the SPAD value and nitrogen content in the leaves hair root length, hair root weight, and hair root number also increased significantly. Plants overexpressing *GmCHI1B1* (OE1B1) showed significantly increased nodule weight, nodule volume, hair root length, hair root weight, and hair root number (see Figure 2b). Plants overexpressing *GmCHI1B2* (OE1B2) did not show significantly effects any phenotypic characteristics. Plants overexpressing *GmCHI1A* (OE1A) were superior to those overexpressing *GmCHI1B1* (OE1B1) in terms of various indices. This suggests that *GmCHI1A* may be a pivotal gene affecting nodulation in soybeans with type II *CHIs* (see Figure 2).

### 3.3. RNA Interference (RNAi) of *GmCHI1s* Genes Disrupts Nodulation and Growth in Soybean

**Figure 3.**
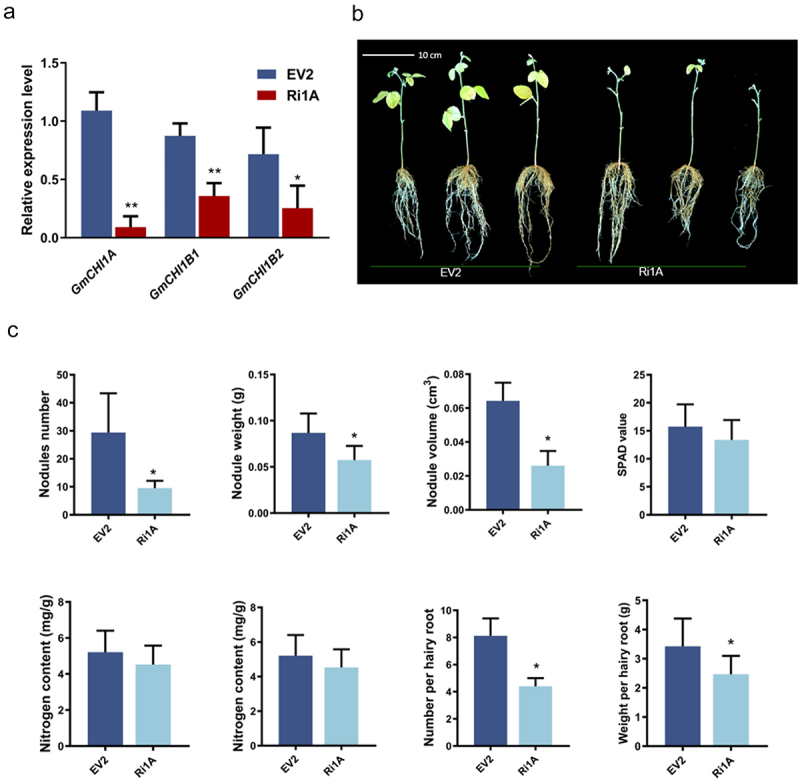
The effect of interfering *GmCHI1A* gene on root nodulation formation in soybean. (a) Relative expression of *GmChI1s* in transformed hairy roots of interfering *GmCHI1A.* (b) phenotype of the transformed hairy roots of interfering *GmCHI1A*. (c) phenotypic comparison between transgenic composited plants with interfering *GmCHI1A* and control (** and *indicate significance at *p* ≤ .01 and *p* ≤ .05 respectively).

To further confirm the effect of *GmCHI1A*, an interference vector for *GmCHI1A* was constructed, and composite plants interfering with *GmCHI1A* (Ri1A) were obtained using the soybean hair-rooting technique. The control group was transfected with the empty vector, pCHF-1301 (EV2). The expression of *GmCHI1B1* and *GmCHI1B2* did not show a compensatory mechanism after interference with *GmCHI1A*; however, both showed a decreasing trend (see Figure 3a). The aboveground portion of Ri1A plants exhibited indications of stunting to some extent, as evidenced by the smaller leaf area, yellowish leaf color, and early leaf abscission (see Figure 3b). The phenotypic data showed that the aboveground biomass (e.g., chlorophyll SPAD value and nitrogen content) and hairy root length of Ri1A showed a decreasing trend compared with the control, although the difference was not significant. The number of hairy roots, weight of hairy roots, number of rhizomes, weight of rhizomes, and volume of rhizomes were significantly lower in Ri1A plants than the control (see Figure 3c). This indicated that interference with the *GmCHI1A* gene affected the formation of soybean nodules and had a certain effect on growth and development (see Figure 3).

### 3.4. *GmCHI1A* Influences the Expression Levels of Genes in the Soybean Nodulation Signaling Pathway

**Figure 4.**
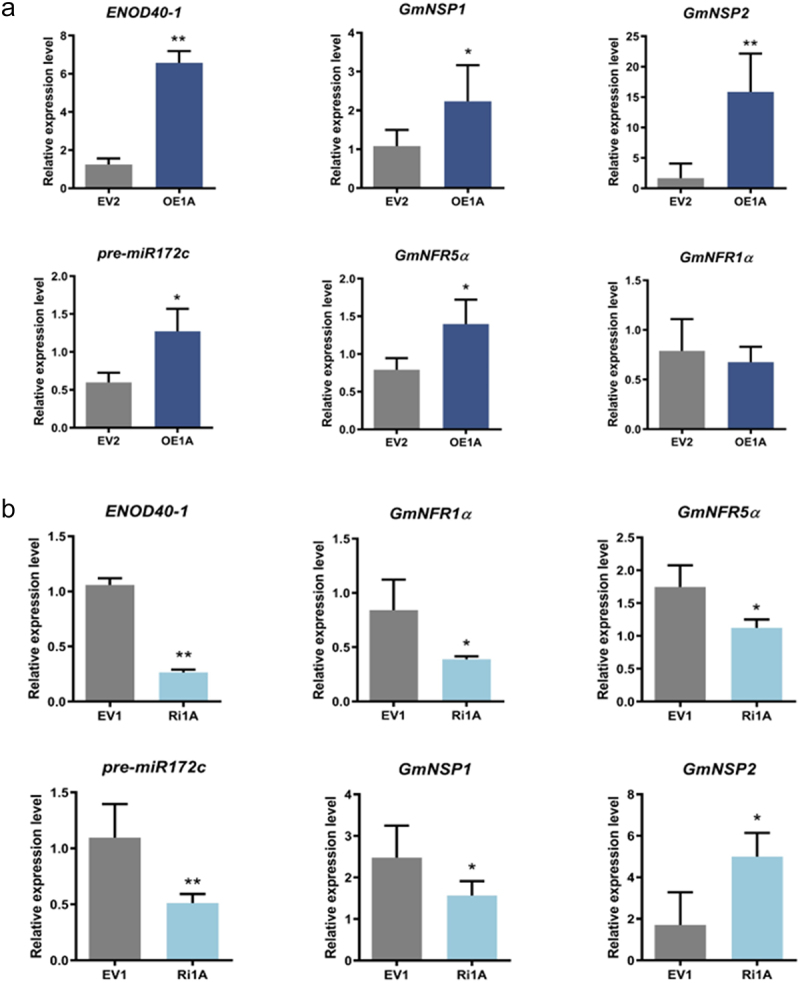
Alterations in *GmCHI1A* expression affect the transcript levels of nodule-specific genes. (a) Relative expression levels of related genes in transgenic roots overexpressing *GmCHI1A*. (b) relative expression levels of related genes in transgenic roots interfering *GmCHI1A*. data are means±sd. Asterisks above the columns represent statistically significant differences (*p < .05, **p < .01; Duncan’s test).

The expression of relevant nodulation pathway genes was detected using qRT-PCR. The results showed that the expression levels of relevant nodulation signaling pathway genes, especially *Early Nodulin 40–1*(*ENOD40–1*), *Glycine max Nodule factor receptor 5α* (*GmNFR5α*), *Glycine max Nodule factor receptor 1α* (*GmNFR1α*), *microRNA172c precursor* (*pre-miR172c*), *Glycine max Nodulation signaling pathway 1* (*GmNSP1*), and *Glycine max Nodulation signaling pathway 2* (*GmNSP2*), were significantly higher in OE1A compared with the control, although no significant difference was observed in the expression of *GmNF1α* (see Figure 4a). The expression of relevant nodulation signaling pathway genes, including *ENOD40–1, GmNF5α, pre-miR172C, GmNSP1*, and *GmNF1α*, was significantly lower in Ri1A than in the control group, whereas the expression of *GmNSP2* was significantly higher in Ri1A (see Figure 4b). Therefore, except for *GmNF1α* and *GmNSP2*, all detected genes on the nodulation signaling pathway were co-expressed with *GmCHI1A*. This suggests that *GmCHI1A* positively regulates the expression of genes related to the nodulation signal transduction pathway, which coincides with the rhizome development and root isoflavone content of the transgenic plants, as previously investigated (see Figure 4).

### 3.5. *GmCHI1s* Regulate Soybean Isoflavone Accumulation

**Figure 5.**
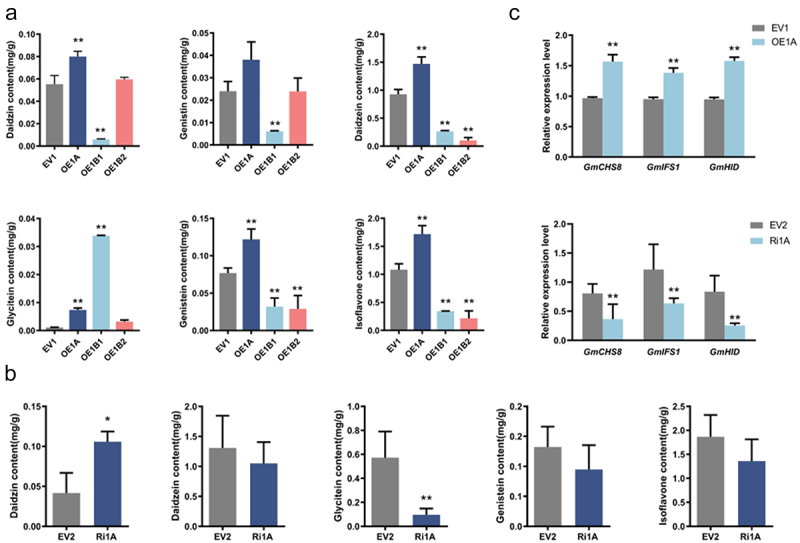
Analysis of isoflavone content and relative expression of related genes in interference and overexpression composite plants. (a, b) quantitative determination of the genistein, genistin, daidztein, daidztin, glycitein content using HPLC methods in overexpression and RNAi transgenic hairy roots. Total isoflavone content is equal to the sum of genistein, genistin, daidztein, daidztin and glycitein contents. Data are means±sd. Asterisks above the columns represent statistically significant differences. (c) qRT-PCR detected the expression level of *GmCHS8, GmIFS1, GmHID* in overexpression *GmCHI1A* and RNAi transgenic hairy roots of composite plants respectively. (*p < .05, **p < .01; Duncan’s test).

The isoflavone content of the soybean roots was determined using HPLC. The data showed that overexpression of each *GmCHI1s* gene had a different effect on the isoflavone content of soybean roots. The daidzin, genistin, daidzein, genistein, and glycitein contents as well as the total isoflavone content were significantly increased in OE1A compared to the control. This suggests that overexpression of the *GmCHI1A* gene in the root can positively regulate isoflavone components. Compared with the control, OE1B1 showed a highly significant increase in glycitein content but a decrease in the rest of the components and total isoflavone content, which may be due to competition for synthesis substrates among the isoflavone components (see Figure 5a). This suggested that *GmCHI1B1* plays a critical role in the synthesis of glycitein. After the interference of *GmCHI1A*, Ri1A showed significant downregulation of glycitein content and a nonsignificant reduction of daidzein, genistein, and total isoflavone contents. The daidzin content of Ri1A showed a certain degree of backfilling (see Figure 5b). Taken together, these results showed that *GmCHI1A* had a positive regulatory effect on all isoflavone fractions and total isoflavone content, *GmCHIB1* positively regulated soybean flavin synthesis but did not affect total soybean isoflavone content, and *GmCHI1B2* had some effect on isoflavone content (see Figure 5).

### 3.6. *GmCHI1A* Regulates Isoflavone Biosynthetic Gene Expression in the Roots

Given that OE1A demonstrated a notable elevation in isoflavone content relative to the control, an investigation was conducted to ascertain whether the expression of genes associated with soybean isoflavone synthesis had undergone alterations in the transgenic roots. To this end, the expression of isoflavone biosynthesis genes, including *Glycine max Isoflavone synthase 1* (*GmIFS1*), *Glycine max Chalcone synthase 8* (*GmCHS8*), and *Glycine max Hydroxyisoflavanone dehydratase* (*GmHID*), was evaluated by qRT-PCR. The expression of *GmIFS1*, *GmCHS8*, and *GmHID* was markedly elevated in OE1A cells relative to that in EV1 cells (see Figure 5c). This finding suggests that the overexpression of *GmCHI1A* in the root lines led to the upregulation of isoflavone synthesis-related genes, thereby increasing the isoflavone content of the root lines. In contrast, the expression of *GmIFS1*, *GmCHS8*, and *GmHID* was significantly reduced in the transgenic hairy roots with interfering *GmCHI1A* (Ri1A) compared to the null control (EV1) (see Figure 5c), suggesting that interfering with *GmCHI1A* genes in the root system exerted an inhibitory effect on the genes related to isoflavone synthesis. Changes in the expression of isoflavone synthesis-related genes in the transgenic plants coincided with changes in isoflavone content (see Figure 5).

## 4. Discussion

BNF by legumes and rhizobia is an efficient, nonpolluting, and sustainable method for plants to obtain nitrogen.^28^ Isoflavones secreted by soybean roots can stimulate signal transduction in symbiotic nodules. This study was focused on CHI, a key enzyme in isoflavone synthesis, to investigate the effect of *CHIs* on isoflavone synthesis and nodule development in soybean.

Isoflavone synthesis is regulated by the phenylpropane metabolic pathway, in which *CHI* is involved in the synthesis of intermediates. Isoliquiritigenin and naringenin chalcones are catalytically synthesized by *CHI* into flavanones (liquiritigenin and naringenin). Liquiritigenin and naringenin are important prerequisites for the synthesis of other metabolites, with liquiritigenin representing a substrate for the biosynthesis of glycitein and daidzein and naringenin representing a substrate for the synthesis of genistein and various anthocyanins.^29^ *CHI* has been studied extensively and its mutants in aster (*Callistephus chinensis*) and carnation (*Dianthus caryophyllus*) lead to yellowish flower petals, while its mutant in *Arabidopsis* has leads to a change in seed coat color.^30^ Overexpression of *CHI* in *Scutellaria baicalensis*, *Glycyrrhiza uralensis*, and *Arachis hypogaea* leads to increased production of flavonoids in hairy roots.^31,32^ In soybean, there are three *CHI1* genes, *GmCHI1A*, *GmCHI1B1*, and *GmCHI1B2*. Expression analysis showed that these three genes were biased in different organs. *GmCHI1A* was expressed at the highest levels in the roots, while *GmCHI1B1* and *GmCHI1B2* were expressed at the highest levels in the seeds and flowers, respectively. We investigated the function of these three genes in the roots and found that *GmCHI1A* promoted the accumulation of all isoflavone components while *GmCHI1B1* promoted the accumulation of glycitein. These results suggest that *GmCHI1s* regulate isoflavone synthesis in soybeans and have evolved to be functionally differentiated.

The nodulation of legume roots is a process in which the host plant interacts with rhizobacteria and involves a complex series of regulatory networks that incorporate many genes. In *M. truncatula*, knockdown of *MtENOD40–1* expression resulted in a 50% reduction in nodule number, suggesting that *ENOD40* is involved in nodule initiation.^33^ Research has confirmed that *NSP1* and *NSP2* interact to form a complex, which is enhanced by Nod factor perception and necessary for the proper development of nodules.^34^ *NF1α* and *NF5α* are two highly related lipo-oligochitin LysM type receptor kinase genes that are presumed to be critical nodulation inducing (Nod) factor receptors.^35,36^ Additionally, *miR172c* has been shown to play an essential role in the control of nodulation through the *GmNINa*–*miR172c*–*GmNNC1* regulatory network.^37^ In this study, *ENOD40–1*, *NF5α*, *miR172c*, *NSP1*, and *NSP2* were significantly increased after overexpression of *GmCHI1A* while *ENOD40–1*, *NF1α*, *NF5α*, *miR172c*, and *NSP1* were significantly decreased after interference with *GmCHI1A*. These results are consistent with those of previous studies. This suggests that *GmCHI1A* is involved in nodule formation and affects the intensity of nodule signaling.

Isoflavonoids are released from plant roots and act as signaling molecules, activating nod operons in compatible bacteria, which induce nodule formation and nitrogen fixation.^38^ Isoflavones contain 12 different isomers in four structural forms (aglycone, glucoside, 6″-O-acetylglucosides, and 6″-O-malonylglucosides). Daidzein, glycitein, and genistein belong to the aglycone type.^39^ In particular, the symbiotic interaction between *Phaseolus vulgaris* roots and *Rhizobium leguminosarum* is regulated by genistein, daidzein, and coumestrol.^40^ In soybeans, the main isoflavonoids perceived by rhizobacteria are daidzein and genistein.^41^ Most isoflavonoids in root secretions early in the growth of soybean plants are soybean daidzein derivatives.^16^ Ramongolalaina suggested that genistein is involved in controlling soybean nodule formation through a QTL analysis of genetic interrelationships between soybean nodule interactions and root-secreted genistein.^42^ In this study, the nodule number and daidzein and genistein content increased in composite plants overexpressing *GmCHI1A* and decreased in composite plants interfering with *GmCHI1A*, this suggests that *GmCHI1A* may influence nodule formation by regulating daidzein and genistein synthesis.

Composite plants overexpressing *GmCHI1B1* also showed significant increases in nodule number, weight, and volume. Unexpectedly, only the glycitein content significantly increased in overexpressing *GmCHI11B1* roots, whereas all other isoflavonoid fractions decreased. Previous studies have shown that glycitein belongs to the same type of aglycone structure as genistein and daidzein. Therefore, glycitein might have functions similar to those of genistein and daidzein. However, glycitein has only been studied for disease resistance and antioxidant purposes^43^ and rarely for nodule development. The results of this study showed that *GmCHI1B1* affected nodule formation by regulating glycitein content. Therefore, whether glycitein can replace genistein and daidzein to promote plant nodule development should be determined.

Nitrogen fixation in soybeans not only provides nutrients to crops, but also contributes to the improvement of crop ecosystems and may provide a feasible method of resolving the multiple societal crises of population, food, energy, and environment. It is of great significance to analyze the molecular mechanism of the soybean-rhizobium symbiosis system, which can aid in the selection of soybean varieties and achieve fertilization requirements and environmental protection. The results of this study showed that *GmCHI1A* and *GmCHI1B1* could be used as genes to enhance nitrogen fixation in soybeans and thus could be applied to improve soybean varieties.

## 5. Conclusion

In this study, *GmCHI1A* was expressed at the highest level in soybean roots, and its overexpression in soybean significantly promoted nodule development. However, interference with *GmCHI1A* in soybean reduced nodules. This demonstrates that *GmCHI1A* is the main effector gene that regulates nodule formation in type II *GmCHIs*. Overexpression of *GmCHI1A* increased the content of all the isoflavone fractions. However, overexpression of *GmCHI1B1* increased the glycitein content, which also promoted nodule development. Therefore, this study is the first to propose that glycitein may affect soybean nodulation.

This study revealed the mechanism by which type II *GmCHIs* affect nodulation in soybeans and screened the main functional genes, providing a theoretical basis for subsequent research on BNF. Glycitein, an isoflavone that affects nodulation in soybeans, was discovered for the first time, providing new ideas for the subsequent enrichment of soybean nodulation signaling pathways.

## Supplementary Material

Supplemental Material
