# Supplementary material for: Functional analysis of type II chalcone isomerase (CHI) genes in regulating soybean (Glycine max L.) nodule formation
Source: GM Crops Food. 2025 Mar 31;16(1):305–17. doi: 10.1080/21645698.2025.2486280 (PMC11970754; doi:10.1080/21645698.2025.2486280)
Supplement: Supplemental Material [file KGMC_A_2486280_SM6464.docx]

1. **Supplemental material**

**
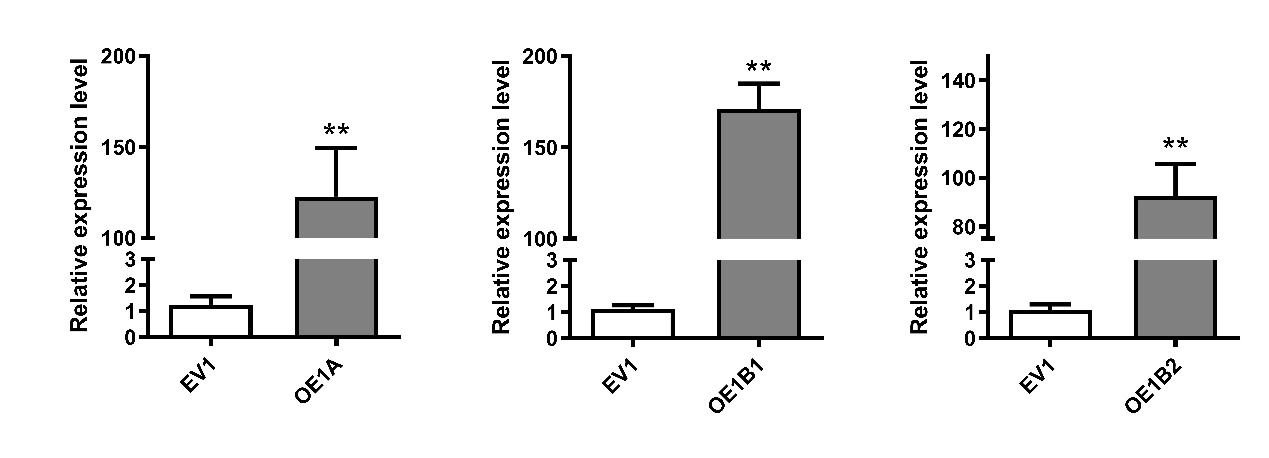
Figure S1.** Expression levels of genes after overexpression

**Table S1.** The primers for cloning and qRT-PCR

| Primer Name | Primer Sequence（5’-3’） |
| --- | --- |
| *GmCHI1A* | F:AGTGATTGTGGTCTCATTCATTTG  R:TTCACCAACTCTAACAGAACTCAAT |
| *GmCHI1B1* | F:AAGTGATTGTGGTCTCATTCATTTG  R:TTCACCAACTCTAACAGAACTCAAT |
| *GmCHI1B2* | F:TAACCATCTTGCCTCT  R:TTGTTGTTCCTGTT |
| *GmActin* | F:GTCCTTTCAGGAGGTACAACC  R:CCACATCTGCTGGAAGGTGC |
| qRT-*GmCHI1A* | F:TCCAGCGGTGGTTACTTCAC  R:GTATTCAGCGCCAGCCAATG |
| qRT-*GmCHI1B1* | F:TCTGTTGGGACTTACAGTGATGA  R:GCCTCCGAAAGTGGCTTGTT |
| qRT-*GmCHI1B2* | F:TTGGGACTTACAGTGAAGCAGA  R:AACGGCCTCCGAGAGTGG |
